# Immediate breast reconstruction with a saline implant and AlloDerm, following removal of a Phyllodes tumor

**DOI:** 10.1186/1477-7819-9-34

**Published:** 2011-03-21

**Authors:** Shirley A Crenshaw, Michael D Roller, Jeffery K Chapman

**Affiliations:** 1 Department of Chemistry, Colorado State University, Fort Collins, CO 80523, USA; 2 Northern Colorado Surgical Associates, Fort Collins, CO 80528, USA; 3 Northern Colorado Plastic Surgery, Fort Collins, CO 80524, USA

## Abstract

**Background:**

Phyllodes tumors are uncommon tumors of the breast that exhibit aggressive growth. While surgical management of the tumor has been reported, a single surgical approach with immediate breast reconstruction using AlloDerm has not been reported.

**Case presentation:**

A 22-year-old woman presented with a 4 cm mass in the left breast upon initial examination. Although the initial needle biopsy report indicated a fibroadenoma, the final pathologic report revealed a 6.5 cm × 6.4 cm × 6.4 cm benign phyllodes tumor *ex vivo*. Treatment was a simple nipple-sparing mastectomy coupled with immediate breast reconstruction. After the mastectomy, a subpectoral pocket was created for a saline implant and AlloDerm was stitched to the pectoralis and serratus muscle in the lower-pole of the breast.

**Conclusions:**

Saline implant with AlloDerm can be used for immediate breast reconstruction post-mastectomy for treatment of a phyllodes tumor.

## Background

Cystosarcoma phyllodes was first described in 1838 by Johannes Müller but was not found to be malignant until 1943 by Cooper and Ackerman [1]. It is now commonly called phyllodes tumor. It is less than 1% of breast tumors and exhibits unpredictable behaviour.

Reports in literature have been focused on surgical approaches to the tumor removal. Although patient assessment prior to tumor removal often includes plans for immediate breast reconstruction, these approaches are rarely reported unless the tumor is classified as giant or is in an adolescent female [2-5]. Usual tumor treatment is wide local excision and simple mastectomy [6-8]. However, there have been few reports on breast reconstruction with phyllodes tumors, especially within the last 10 years. Because of the fast growth rate of these tumors, a greater than a 1 cm negative margin is preferred with tumor removal and a mastectomy may have to be performed to prevent local reoccurrence. Breast reconstruction usually consists of a transverse rectus abdominis musculocutaneous (TRAM) flap or a latissimus dorsi (LD) musculocutaneous flap as in other breast cancers.

Here we report a single surgery that includes reconstruction of the breast immediately post-mastectomy using a saline implant and AlloDerm. AlloDerm is becoming increasingly popular for immediate breast reconstruction. It is a viable option for athletic or thin women for whom TRAM or LD is not possible. A submuscular pocket can be created for a breast implant and AlloDerm is used to give lower-pole fullness. It helps fill the breast flap when subcutaneous tissue is limited and supports the breast implant which can have issues such as rippling or bottoming out [9,10].

## Case presentation

The patient was a 22-year-old African American female who presented with a left breast mass. The mass had been present for at least 3 months. The left breast was larger and leaked a clear fluid. Upon initial examination, the patient's primary care provider observed a 4 cm mass. The patient also experienced pain down the left arm which was reported about a week after the initial exam. Her aunt on her father's side had been treated for breast cancer at 39. She had no past medical or surgical history, did not use tobacco, and menarche occurred at 11 years of age.

**Figure 1:**
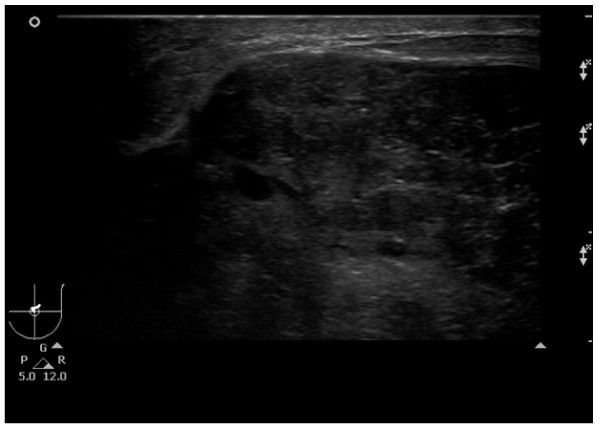
**Ultrasound of benign phyllodes tumor in left breast**.

Ultrasound of the left breast (Figure 1) revealed a 9 cm × 9 cm × 4.5 cm hypoechoic mass centered at the 12-1 o'clock area. The anterior of the mass appeared to be within 1 cm of the skin and the posterior was on the pectoral muscle. The echotexture varied from hypoechoic to isoechoic and there are small cystic areas within the mass. There were no other masses identified in the left breast or the left axilla. The mass was deemed suspicious for malignancy and the assessment was Bi-RADS 4b. The patient was sent for surgical consultation.

Upon surgical consultation a core needle biopsy was performed with a 22-gauge needle; four biopsies were obtained. The biopsies were consistent with a fibroadenoma, indicating a benign tumor, which was consistent with the presentation of most cystosarcomas in core needle biopsies [11,12].

The patient underwent a simple mastectomy with immediate breast reconstruction. Because the tumor volume was approximately two thirds of the breast and lumpectomy would result in poor cosmetic outcome, simple mastectomy with nipple-areola complex (NAC) preservation was performed on the patient. Standard breast reconstruction usually consists of a transverse rectus abdominis musculocutaneous flap or a latissimus dorsi musculocutaneous flap. However, the patient did not have adequate fatty tissue at the abdomen for the TRAM procedure and the patient did not want the large scar across the abdomen that would result. The plastic surgeon thought LD was the better choice because it would provide a "living" breast but this still required a small implant. The patient opted for a saline implant with AlloDerm to the mastectomy site and a mastopexy (breast lift) or mastopexy and implant to the other breast for symmetry. Because breast reconstruction was coupled with mastectomy using AlloDerm, there was no need for a TRAM or LD surgery which reduced scarring and the patient's overall recovery time.

The simple mastectomy consisted of an incision along the mammary crease. Dissection of the breast was carried out by cutting down the anterior pectoral fascia and dissecting to approximately 1 cm from the clavicle. Medial dissection was carried out to the left lateral border of the sternum and lateral dissection was carried over to the anterior border of the latissimus dorsi muscle. A bovie electrocautery was used to create a superior flap of the entire left breast.

Next, breast reconstruction was performed. At the level of the inframammary fold, the pectoralis muscle was divided from 4 to 8 o'clock and a subpectoral/subserratus pocket was made using an electrocautery. At the mastectomy site, the superior pole of the breast flap was thicker than the inferior pole. Therefore, the superior breast tissue was laterally divided and sutured under the skin and to the pectoralis muscle with 3-0 Monocryl. This smoothed out the contour of the superior pole. AlloDerm, used to give more lateral fill, was sutured to the inframammary fold and to the part of the pectoralis muscle with 3-0 polydioxanone. The saline implant was then inserted into the pocket. The flap was advanced down, sutured into place, and the implant was filled. A drain was inserted and the incision was closed with Monocryl and Dermabond. For symmetry, the other breast underwent vertical mastopexy and positioned by making the superior border of the areola at the same level as the other breast.

**Figure 2:**
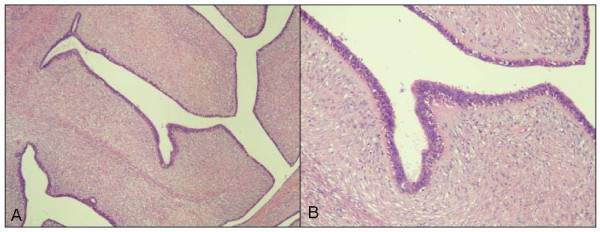
**A. Image of leaf-like cystic ducts projected into the stroma**. B. Image of one cystic duct.

**Figure 3:**
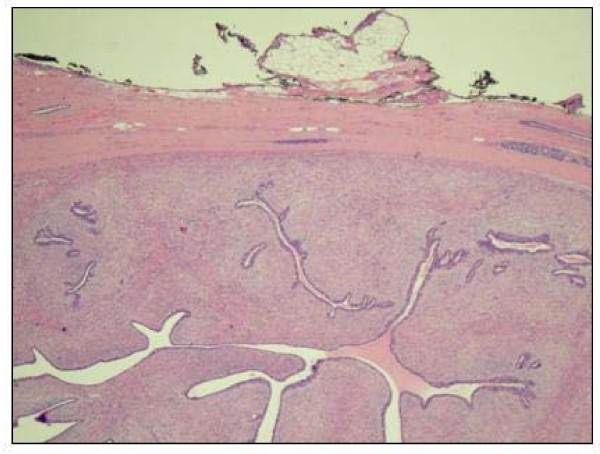
**Negative margin of resection for the benign phyllodes tumor**.

Grossly, the excised encapsulated mass measured 6.5 cm × 6.4 cm × 6.4 cm. The surface was tan with a whorled appearance. Pathologic findings were consistent with a benign phyllodes tumor displaying large leaf-like projections surrounded by uniform stroma (Figure 2a and 2b) and black-inked margins of resection were negative (Figure 3).

**Figure 4:**
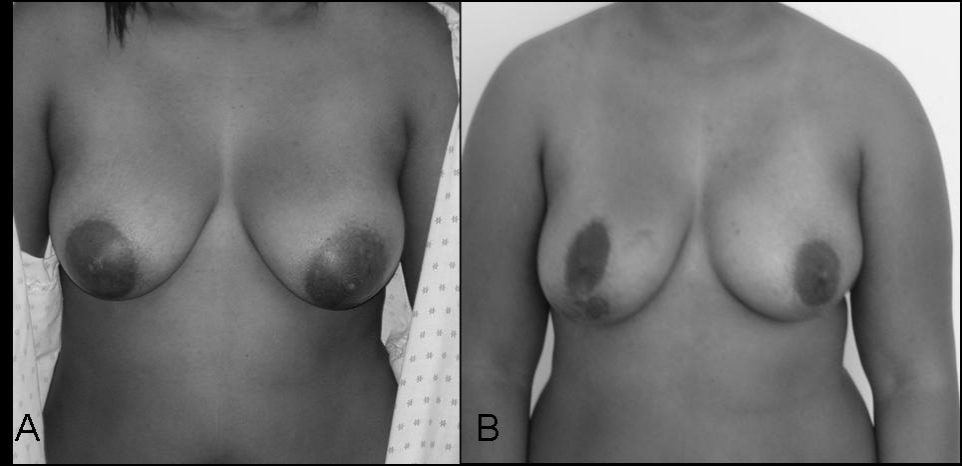
**A. Preoperative view of phyllodes tumor in left breast**. B. Postoperative view after 4 years with nipple-sparing mastectomy.

There were no postoperative complications and hospital stay was 24 hrs. The patient is currently 41 months post-surgery and has not had local reoccurrence. The patient is very pleased with the cosmetic outcome (Figure 4) and feels that immediate reconstruction was helpful in reducing emotional distress from the diagnosis and surgery.

## Discussion

Phyllodes tumor is a disease of the epithelial and stroma tissue in the breast. It is classified as benign, borderline, and malignant. Malignant tumors have high stroma cellularity and tend to be permeative whereas benign tumors have low stroma cellularity and are circumscribed [6,7,13,14]. Borderline tumors cannot be distinguished between the two because of the uncertainty of their behavior. These tumors can be encapsulated and their size typically ranges from 1-45 cm [6]. These tumors, when discovered, are usually large from their aggressive growth rate and at beginning stages, cause virtually no pain or other symptoms [15]. Larger tumors can result in nipple discharge, deformity of the skin, pain from the tumor weight, or pain from the impact on nerves [8,15]. There is no correlation between size and tumor malignancy [7,16].

Treatment for the disease usually involves wide local excision with negative margins greater than 1 cm [6-8]. If there is poor tumor to breast size, simple mastectomy is recommended. There have been a few cases reported of benign tumors metastizing but this is very rare [7]. These tumors tend not to metastasize to the axillary lymph nodes but more commonly to bone, lungs, and liver [6-8]. Adjuvant radiotherapy and chemotherapy generally are not used because the benefit of these therapies is unclear [6,7,17]. However, if the margins are less than 1 cm and there is chest wall invasion, adjuvant radiotherapy should be strongly considered [6].

As discussed in this report, breast reconstruction can be performed immediately after tumor removal. Although immediate breast reconstruction is oncologically safe to perform after mastectomy [9], Mortenson and co-workers found wound healing complications increased from 8.3% to 22.2% [18]. In three breast reconstruction groups, the tissue expander/implant, TRAM, and LD group, the site complications were 11.5%, 33%, and 83% within their own group, respectively.

In breast reconstruction, the NAC is preserved, if possible, for the best cosmetic outcome. It is still controversial to preserve the NAC when the tumor is cancerous and centrally located because it is unclear if the NAC is involved with breast cancer [19]. If removed, the NAC can be reconstructed. Common techniques to reconstruct the NAC are skin graft and tattoo but it is difficult to obtain nipple symmetry and reconstructed nipples often have poor nipple projection, color match, shape, and texture [19].

There are four main incisions for nipple-sparing mastectomy. The superior or inferior periareolar with lateral extension, transareolar with perinipple and lateral-medial extension, transareolar and transnipple incision with medial and lateral extension, and mammary crease that is inferior or lateral. The superior or inferior periareolar with lateral extension allows good exposure for tumor removal but may compromise blood supply to the periphery of the flap and areola [19,20]. The transareolar with perinipple and lateral-medial extension also provides good exposure and reduces the risk of ischemia to the lower portion of the areola. However, care must be taken not to divide the perinipple artery from the breast parenchyma causing perinipple scaring resulting in downward nipple projection [19,20]. The transareolar and transnipple incision with medial and lateral extension provides good exposure to the lactiferous ducts for dissection and good vascularity to the areola and nipple, but the apical portion of the nipple may still suffer form ischemia or necrosis [20]. The mammary crease approach, inferior, was used here. The scar is the least visible and the skin flap vascularization is supported by superior and medial vessels [19,20]. Vascularization of the nipple and areolar are not disturbed with this incision [20]. This incision is best for smaller breast with low ptosis as it may be difficult to reach parasternal and subclavicular areas of the breast for tumor removal. All incisions have equal risk of necrosis but women under 45 years of age had a higher rate of NAC viability [21].

Since immediate breast reconstruction is being used with increasing frequency, more surgical approaches are needed for fast recovery. AlloDerm is an acelluar dermal matrix from human cadaver skin. The skin has no cellular components and, for this reason, rejection is not an issue [9]. The use of AlloDerm in breast reconstruction has many advantages. It can be used off the shelf which reduces operation time [9]. If there is not enough subcutaneous tissue to fill a skin flap, AlloDerm can be used to fill out the inferior pole of the breast. AlloDerm also helps visually to reduce rippling, stark contours, and bottoming out seen with larger implants [9,10]. It also shortens recovery time. Hospital stay for AlloDerm/implant breast reconstruction was found to be an average of 48 hours [22]. It is safe to use and provides another option for immediate breast reconstruction.

## Conclusions

Management of phyllodes tumors has many challenges which need to be addressed on a case by case basis. In this case, a simple mastectomy was the best option for a young patient with a comparatively large tumor mass. Although LD with an implant was thought to be the best choice for breast reconstruction, the patient opted for just a saline implant with AlloDerm and mastopexy for symmetry. The cosmetic outcome was good. Therefore implant reconstruction with AlloDerm should also be considered along with LD and TRAM if the patient wants minimal scarring and reduced recovery time.

## Consent

Written informed consent was obtained from the patient for publication of this case report and accompanying images. A copy of the written consent is available for review by the Editor-in-Chief of this journal.

## Competing interests

The authors declare that they have no competing interests.

## Authors' contributions

SAC prepared the manuscript. MDR provided patient medical records and carried out the mastectomy. JKC carried out the breast reconstruction. All authors read and approved the final manuscript.

## Authors' information

SAC is a Ph.D. candidate in the Department of Chemistry at Colorado State University.
